# CD55 is a HIF-2α marker with anti-adhesive and pro-invading properties in neuroblastoma

**DOI:** 10.1038/oncsis.2016.20

**Published:** 2016-04-04

**Authors:** F Cimmino, M Avitabile, L Pezone, G Scalia, D Montanaro, M Andreozzi, L Terracciano, A Iolascon, M Capasso

**Affiliations:** 1 Dipartimento di Medicina Molecolare e Biotecnologie Mediche, Università degli Studi di Napoli ‘Federico II', Naples, Italy; 2 CEINGE Biotecnologie Avanzate, Naples, Italy; 3 Dipartimento di Medicina, Scuola di Medicina e Chirurgia, Università degli Studi di Verona, Verona, Italy; 4 Institute of Pathology, University of Basel, Basel, Switzerland

## Abstract

CD55 has been revealed to have an important role in tumor genesis, and presence of small populations of cells with strong CD55 expression would be sufficient to predict poor prognosis of several tumors. In our study we revealed that CD55 is a novel target of hypoxia-inducible factor HIF-2α in neuroblastoma (NB) cells. We show that HIF-2α expression is sufficient to sustain stem-like features of NB cells, whereas CD55 protein upon HIF-2α expression contributes to growth of colonies and to invasion of cells, but not to stemness features. Interestingly, in NB tissues, CD55 expression is limited to quite a small population of cells that are HIF-2α positive, and the gene expression of CD55 in the NB data set reveals that the presence of CD55^high^ affects prognosis of NB patients. The functional characterization of CD55-positive populations within heterogeneous NB monoclonal cell lines shows that CD55 has pro-invading and anti-adhesive properties that might provide the basis for the ability of solid tumors to survive as microscopic residual disease. The easy accessibility to CD55 membrane antigen will offer the possibility of a novel antibody approach in the treatment of recurrent tumors and will provide a ready target for antibody-based visualization in NB diagnosis and prognosis.

## Introduction

Neuroblastoma (NB) is a childhood tumor derived from precursor or immature cells of the ganglionic lineage of the sympathetic nervous system (SNS).^1^ The clinical NB hallmark is the large heterogeneity, with the likelihood of tumor progression varying widely according to stage, age at diagnosis and anatomical site. Some NBs could undergo spontaneous regression that is partially regulated by developmentally programmed neuronal cell death and/or neuronal differentiation.^2^

The stage of disease as formulated in the International Neuroblastoma Staging System is considered a marker of tumor burden and underlying tumor biology. Children >18 months with stage 4 (metastatic) disease are at high risk for death from refractory disease. In contrast, children with localized tumors (stage 1–2–3) are almost always cured with radiation or chemotherapy.^3^

NB as a solid tumor is a condition dictated by the proliferation of a single clone of immature cells that might sustain NB formation and growth as a result of acquired additional genetic abnormalities.^4^ The small population of immature cells has features of cancer-like stem cells that are enhanced by restricted oxygen conditions.^5^ Of note, cancer stem cells (CSCs) are critically dependent on the hypoxia-inducible factors HIF-1α and HIF-2α (HIFs) for survival, self-renewal and tumor growth.^6^ Interestingly, HIF-1α is expressed in both CSCs and non-stem cancer cells upon induction of hypoxia, whereas HIF-2α is highly induced only in CSC populations and promotes stem-like phenotype and increases tumorigenic potential in non-stem cancer cells.^7^ In NB tumor-initiating cells HIF-2α expression maintains an undifferentiated state and HIF-2α knockout in NB samples impairs tumorigenesis and leads to a less aggressive/more differentiated phenotype.^8^ Unraveling HIF-2α molecular targets in NB tumors might provide accessible drug targets in non-well-oxygenated areas and will give the possibility of targeting the heterogeneous pool of cells with stem-like properties.

CD55 is a glycosylphosphatidylinositol-anchored protein that inhibits the activation of the complement pathway. CD55, which is expressed in cells exposed to the complement system, binds to C3 convertases generated from both classic and alternative complement pathways, prevents C3b deposition and inhibits the formation of membrane attack complex.^9^ As glycosylphosphatidylinositol (GPI)-anchored protein, CD55 is either bound to the cell membrane or released from the membrane into the microenvironment.^10^

In tumors a subpopulation overexpressing CD55 represents an important mechanism of immune escape adopted to avoid recognition by the immune system or of survival from antibody-mediated immunotherapy.^11^ CD55 expression has been detected in clinical specimens from various kinds of malignant tumors. CD55 expression is higher in prostatic carcinoma,^12^ gastric adenocarcinoma^13^ and lymphoma,^14^ and is an independent factor of poor prognosis in colon^15^ and breast cancer.^16, 17^ Moreover, CD55 knockdown or CD55 low expression reduced tumorigenicity of prostatic adenocarcinoma and breast cancer in immunodeficient mice.^12, 18^ Interestingly, CD55 expression varies among monoclonal cell lines. For example, in breast cancer cell lines CD55-^high^ population could be isolated with cancer stem cell features such as enhanced apoptosis resistance and enhanced colony formation.^19^ These findings suggest that, beyond the inhibition of complement attack, CD55 might have an important role in tumorigenicity of cancer cells, which remains to be investigated.

In our study we revealed that CD55 is a novel target of HIF-2α in NB cells and that CD55 expression downstream of HIF-2α expression is necessary for tumor cell growth and invasion. Moreover, we dissected the role of CD55 in providing data that support the anti-adhesive and pro-invading properties of CD55 molecule antigen in NB cells *in vitro*. These findings provide a new therapeutic marker that is easy to target with monoclonal antibody, which might offer an advantage to therapy with HIFs inhibitors.

## Results

### CD55 and HIF-2α immunohistochemistry co-staining on NB tissue microarray

**Figure 1:**
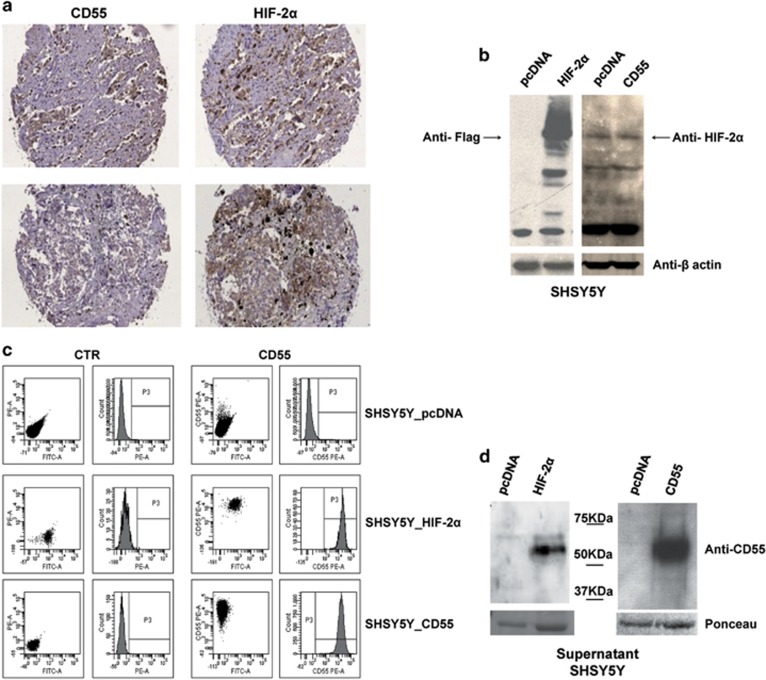
CD55 is a HIF-2α marker. In **a**, one representative example of a total of 92 NB cells positively co-stained for CD55 and HIF-2α is shown. The left picture shows NB section stained with anti-CD55 antibody, the right picture shows a slide of the same section stained with anti-HIF-2α antibody. In western blotting analysis (**b**) HIF-2α protein expression in SHSY5Y HIF-2α stably expressing cells (HIF-2α) and SHSY5Y CD55 stably expressing cells (CD55) is verified. Anti-Flag antibody has been used to detect the ectopic expression of HIF-2α in HIF-2α cells, and anti-HIF-2α antibody has been used for the detection of endogenous HIF-2α protein in CD55 cells. Cells stably transfected with empty vector were used as control cells (pcDNA). β-Actin was used as loading control. FACS analysis (**c**) shows the expression of CD55 protein on the cell surface in both cell clones (HIF-2α, CD55) and pcDNA cells. Cells unstained were used as blank control (CTR). Western blotting (**d**) on supernatant from both cell clones and pcDNA cells shows CD55 secreted form expression in overexpressing cell clones (HIF-2α, CD55). The data were normalized with respect to ponceau.

CD55 protein expression was investigated in NBs. We hybridized a tissue microarray containing 92 NBs at diverse stages of disease with a CD55-specific antibody. We observed not homogeneous but limited CD55 staining to small cell populations within tumors (Figure 1a). CD55 might be regulated by HIF, as a HIF-functional binding site has been identified on the CD55 promoter.^20^ In addition, in NB, HIF-1α protein staining is related to hypoxic areas, whereas HIF-2α protein staining is extended to perivascular areas and is a sign of poor prognosis.^21^ To verify whether CD55 correlates with HIF-2α staining we hybridized the tissue microarray (TMA) with HIF-2α-specific antibody (Figure 1a). We observed small populations of tumor cells co-stained with HIF-2α and CD55 antibodies. The cell populations expressing CD55 at high levels showed high levels of HIF-2α, and cell populations expressing CD55 at low levels showed low levels of HIF-2α. The protein levels of CD55 and HIF-2α were significantly correlated (Spearman's *r*=0.27; *P*-value=0.009). In the analyzed samples we also detected HIF-2α-positive cells without CD55 positivity, but CD55 positivity implicates HIF-2α protein staining. These findings suggest CD55 as a novel HIF-2α marker in a subset of cells within solid tumors suitable for antibody targeting.

### CD55 is a univocally HIF-2α-related target

To verify whether CD55 is a HIF-2α marker we generated NB cells stably expressing the transcription factor HIF-2α by transfecting SHSY5Y cells with pcDNA expression vector coding HIF-2α (HIF-2α) or with empty vector (pcDNA). HIF-2α protein is stable under normoxic conditions *in vitro* as previously described.^22^ As shown by western blotting (Figure 1b) Flag protein detection confirms ectopic HIF-2α protein overexpression, which shows multiple degradation products under normoxic conditions. This HIF-2α overexpression is enough to influence the transcription of the target genes downstream of HIF-2α, as shown in Supplementary Figure 1.

In SHSY5Y_HIF-2α-overexpressing stable cells, CD55 is strongly enhanced in normoxia (100% positivity), whereas the cells transfected with empty vector SHSY5Y_pcDNA do not show detectable CD55 expression levels as assessed by fluorescence-activated cell sorting (FACS) analysis (Figure 1c). Additionally, SHSY5Y was transfected with pcDNA-expressing vector coding CD55 (CD55), and CD55 overexpression was verified by FACS analysis (100% positivity; Figure 1c). In SHSY5Y_CD55-overexpressing stable clones we observed that CD55 does not influence the expression of HIF-2α protein (Figure 1b).

Together these findings show that CD55 might be a direct/indirect target of HIF-2α under normoxic conditions, thus excluding the possibility that the O_2_ concentration might influence its expression levels and can prove a direct transcription regulation of CD55 by HIF-2α and not vice versa. The direct regulation of HIF-2α on CD55 expression was also assessed in several NB cell lines that, upon mRNA silencing of the HIF-2α gene (*EPAS1*), show a decreased expression of CD55 mRNA (Supplementary Figure 2). Moreover, by western blotting analysis on supernatant from SHSY5Y_HIF-2α and SHSY5Y_CD55 stable clones we detected the soluble CD55 protein released by the cells in the medium (Figure 1d). The absence of CD55 in the medium from cells transfected with empty vector suggest that the increased secretion is due to CD55 overexpression in both cell clones.

### HIF-2α enhances stem-like properties of NB cells, whereas CD55 expression is necessary for colony growth and invasion

**Figure 2:**
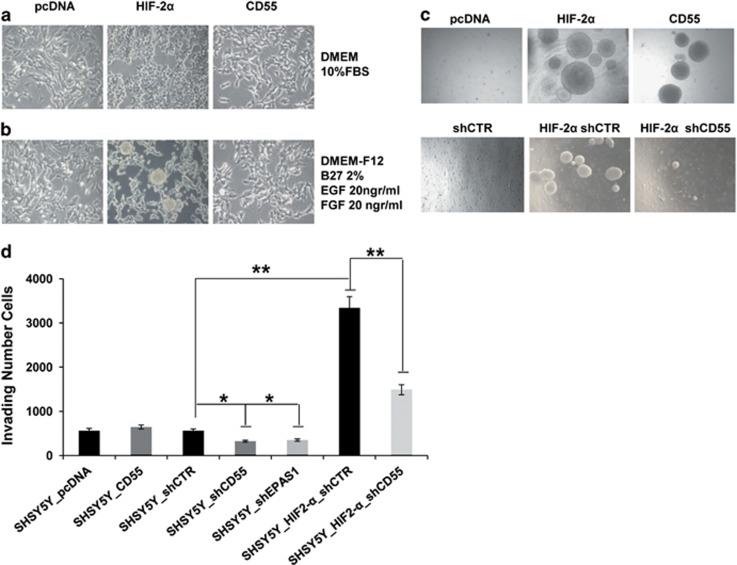
CD55 is necessary for colony growth and invasion, not for stem-like features. The SHSY5Y_HIF-2α, CD55 and pcDNA cell phenotype grown in DMEM medium (**a**) or grown in neurobasal medium (**b**) is shown. The results are shown as the mean of three experiments. Photos in **c** show soft agar assay performed on SHSY5Y_pcDNA clones and on SHSY5Y_HIF-2α and _CD55-overexpressing clones; soft agar performed on SHSY5Y_HIF-2α cells infected by lentiviral delivery of hairpin RNA directed against CD55 (HIF-2α shCD55) or infected by lentiviral delivery of non-silencing hairpin RNA (HIF-2α shCTR) and on SHSY5Y_pcDNA cells infected by lentivirus-mediated delivery of non-silencing hairpin RNA (shCTR). Invasion assay was performed on the same stable clones and on SHSY5Y_shCD55 and shEPAS1 cells. The number of invading cells for each experimental point is shown in the graph bar (**d**). The data are reported as the mean of three experiments (*P⩽0.05).

HIF-2α overexpression notably enhances phenotypic changes of SHSY5Y cells (Figure 2a). As observed, SH5YSY HIF-2α-overexpressing clones show a small cell body without neuritis, suggesting an undifferentiated effect mediated by HIF-2α protein. To assess the undifferentiated/immature phenotype we checked the gene expression of neuronal and stem markers. As shown in Supplementary Figure 3 the expression of neuronal markers is significantly reduced in overexpressing HIF-2α clones compared with control cells, whereas the stem marker expression increases. In contrast, the overexpression of CD55 in SHSY5Y does not change the cell phenotype and the expression of stem markers (Figure 2a; Supplementary Figure 3).

In order to explore the ability of HIF-2α to promote NB stem-like cell properties in normoxia, the cells were grown in neurobasal medium (Figure 2b). Upon 2 days of culture we observed neurospheres in the medium of HIF-2α cells but we did not observe the same phenotype in CD55 cells. The silencing of CD55 gene expression in HIF-2α cells was not enough to rescue the HIF-2α cell stem-like phenotype in neurobasal medium (data not shown). We suppose that HIF-2α enhances stem-like properties of NB cells but its downstream target CD55 is not sufficient to promote the stem-like phenotype.

To elucidate the biological changes induced by HIF-2α or CD55 protein in SH-5YSY-overexpressing cells anchorage-independent growth and invasion assay was performed (Figures 2c and d). The HIF-2α-overexpressing clones are able to grow in soft agar assay as spheres reaching 300 μm in dimension. Interestingly, we observed that the spheres detached from the agar and grow as fluctuant cell spheres in the medium. The SHSY5Y_CD55-overexpressing stable clones compared with empty vector-transfected cell clones (pcDNA) show the same phenotype of HIF-2α cells in soft agar assay. As HIF-2α is not overexpressed or regulated in CD55 cells we suppose CD55 antigen has a key role in determining the colony growth. These findings are supported by the rescue experiment, whereas the HIF-2α cells lose their ability to growth as fluctuant spheres in soft agar once CD55 gene expression is silenced (HIF-2α shCD55), compared with unsilenced HIF-2α cells (HIF-2α shCTR; Figure 2c; Supplementary Figure 4a). In invasion assay CD55 cells do not show an increased ability to invade the collagen-coated transwell with respect to CD55-nonoverexpressing cells (SHSY5Y pcDNA). In contrast, HIF-2α cells (HIF-2α shCTR) have stronger ability to invade the collagen-coated transwell with respect to SHSY5Y shCTR cells (*P*-value=0.004), which effect was in part rescued when we silenced the expression of CD55 in the HIF-2α-overexpressing clones (HIF-2α shCD55; *P*-value=0.001; Figure 2d; Supplementary Figure 4b). Moreover, the invasion of SHSY5Y cells is impaired by the silencing of endogenous HIF-2α (shEPAS1; *P*-value=0.05) or CD55 (shCD55; *P*-value=0.04), suggesting that both proteins are involved in cell motility. On the basis of these data we can establish that CD55 dependently from HIF-2α expression is necessary to support the migratory/invading ability of tumor cells but is not sufficient to promote stem features.

### CD55^+^ cell subpopulation in NB cell lines enhances colony formation and invasion ability

**Figure 3:**
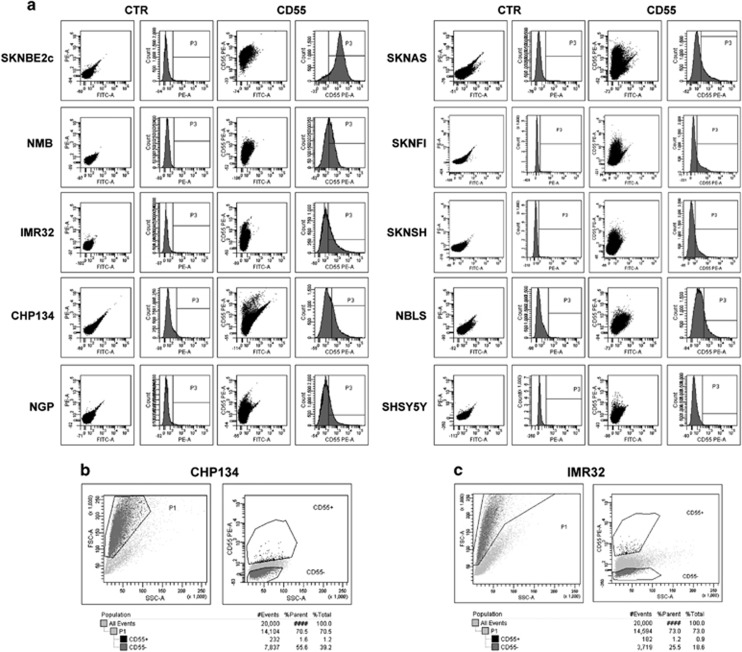
CD55 cell surface staining and CD55 cell sorting in NB cell lines. CD55 surface staining was performed by FACS analysis on several NB cell lines. In the graph the number of CD55-positive cells in P3 is shown (%) (**a**). CHP134 (**b**) and IMR32 (**c**) cells were sorted as CD55^+^ and CD55^−^ by FACS and the gates are shown. Unstained cells were used as blank control to evaluate each antibody and cellular autofluorescence background (CTR). The data were confirmed by three independent experiments.

**Figure 4:**
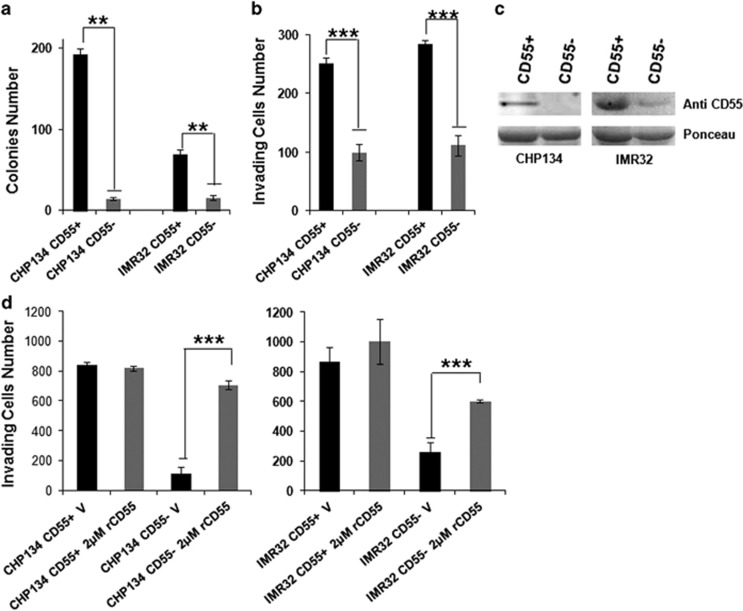
CD55^+^ cell subpopulation shows enhanced colony formation and invasion ability with respect to CD55^−^ cell subpopulation in NB cell lines. Soft agar assay and invasion assay were performed on CHP134 or IMR32 CD55-sorted cells (CD55^+^, CD55^−^). For soft agar assay, colony number is shown in the graph (**a**); for invasion assay invading cell number is shown in the graph (**b**). Antibody anti-CD55 protein detected CD55 secreted form in the medium of CD55-sorted cells by western blotting analysis. Ponceau staining was used as equal loading control (**c**). Invasion assay was performed on CHP134 or IMR32 CD55-sorted cells (CD55^+^, CD55^−^) in the presence of 2 μg human recombinant CD55 (rCD55) (2 μg) or vehicle (V) (**d**). The data are reported as the mean of three experiments (*P⩽0.05, **P⩽0.005, ***P⩽0.0005).

CD55 antigen expression was analyzed in several NB human cell lines by FACS. As shown in Figure 3a, the percentage of cell fraction positive for CD55 is 99.7% in SKNBE2c and is relatively low in other cell lines. It is evident that CD55 antigen marks a constant cell subpopulation in NB cell lines. Moreover, different cell lines show diverse signal intensity on the NB cell surface, dictated by mean fluorescence intensity (MFI; Supplementary Figure 5a). We sought to determine whether the CD55^+^ cell subpopulation might promote the aggressive behavior of the heterogeneous tumor cell lines. To this end we chose CHP134 and IMR32 NB cell lines with similar CD55 surface percentage and different CD55 MFI. The proportion of cells positive for CD55 was ~29.6% (CHP134) and 34.1% (IMR32) and the MFI was ~608 (CHP134) and 191 (IMR32). CHP134 and IMR32 NB cell lines were labeled with anti-CD55 antibody and sorted separately as CD55^+^ and CD55^−^ cells. For cell sorting, CD55^+^ cells were gated in comparison with a negative control (unstained cells) and the sort precision was set on purity (Figures 3b and c). The ability to form colonies of CHP134/IMR32 CD55^+^ and CD55^−^ was assessed in soft agar assay (Figure 4a; Supplementary Figure 5b). The number of colonies of CD55^−^ cells was significantly lower than that of CD55^+^ cells (CHP134, *P*-value=0.001; IMR32, *P*-value=0.04). The invasive ability of CHP134/IMR32 CD55^+^ and CD55^−^ cells was assessed in two-dimensional invasion assay (Figure 4b; Supplementary Figure 5c). The number of CD55^−^ cells able to invade a collagen-coated support was significantly lower than that of CD55^+^ cells for both cell lines (CHP134, *P*-value=1 × 10^−7^; IMR32, *P*-value=1 × 10^−9^). The colony growth and invasion data prove that CD55^+^ cells can promote the aggressiveness of the CHP134 and IMR32 cell lines.

### CD55 secreted form has a paracrine effect on NB cell invasion

We have described that the small CD55^+^ cell subpopulation within a heterogeneous NB cell line shows aggressive tumor features with respect to CD55^−^ cell subpopulation. We have also shown that CD55 is secreted in the medium of HIF-2α or CD55 cells. Here we verified that CD55 is released from CHP134 CD55^+^ cells but not from CHP134 CD55^−^ cells in the medium, confirming that CD55 is overexpressed as membrane antigen and as secreted form. A faint CD55 band is visualized in IMR32 CD55^−^ cells, which may secrete CD55 but do not express CD55 on the membrane (Figure 4c). Our hypothesis is that the great amount of CD55 released by the small CD55^+^ cell subpopulation has an autocrine effect on CD55^+^ cells and a paracrine effect on CD55^−^ cells by leading to the activation of downstream pathways involved in tumor malignant transformation. To assess the pro-invading/migratory ability of secreted CD55, we performed invasion assay of CD55^+^ cells and CD55^−^ cells in the presence of 2 μg human recombinant CD55 protein (Abcam AB168708, Cambridge, UK) in the medium (Figure 4b; Supplementary Figure 5d). As expected, CD55^−^ cells in the presence of recombinant CD55 protein (rCD55) increase their migratory/invading ability with respect to CD55^−^ vehicle-treated cells (CHP134, *P*-value=1 × 10^−9^; IMR32, *P*-value=1 × 10^−4^). In contrast, CD55^+^ cells do not increase their migratory ability in the presence of rCD55 probably because CD55 secreted protein is sufficient to provide a migratory effect on the cells.

### CD55^+^ cell subpopulation in NB cell lines impairs adhesion ability

**Figure 5:**
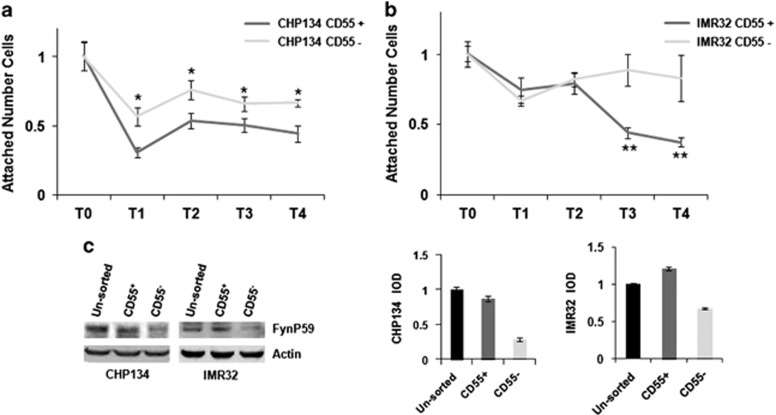
CD55^+^ cell subpopulation shows impaired cell adhesion with respect to CD55^−^ cell subpopulation. Adhesion assay was performed on CHP134- (**a**) and IMR32- (**b**) sorted cells (CD55^+^, CD55^−^). (T0) shows the attached number of cells measured after seeding on the collagen-coated surface of a 96-multiwell. The number of attached cells was measured at 1 h (T1), 2 h (T2), 3 h (T3) and 4 h (T4) from cell seeding. The expression of Fynp59 protein was detected by western blotting in the unsorted (CHP134, IMR32) and sorted cells (CD55^+^, CD55^−^). The bands were quantified by densitometry. The bar graphs show integral optical density (IOD) value for each band, normalized with respect to β-actin expression (**c**). The data are reported as the mean of three experiments (*P⩽0.05, **P⩽0.005, ***P⩽0.0005).

CD55 appears to function as an anti-adhesive surface glycoprotein that regulates the rate of polymorphonucleated cell migration across the apical epithelial membrane.^20^ The cell-to-extracellular matrix or cell-to-cell interaction is necessary for the establishment of the primary site of the tumor, but cells through their migration toward a secondary tumor site lose this function. CD55 antigen enhances the cells' soft agar growth and invasion as described above. We sought to determine whether the aggressive phenotype determined by CD55 antigen might be ascribed to its anti-adhesive properties of reduced cell attachment to a substrate. The attachment assay employs a colorimetric detection of bound cells to measure the contacts between a cell and a specific adhesive substrate: collagen (Figures 5a and b) or fibronectin (data not shown). As shown by the colorimetric assay, the CHP134 CD55^−^ cells show adhesive ability as proved by the major cell numbers attached to the substrate with respect to CHP134 CD55^+^ cells (*P*-value⩽0.05; Figure 5a), and the IMR32 CD55^−^ cell subpopulation shows adhesive ability with respect to the IMR32 CD55^+^ cell subpopulation (*P*-value⩽0.005 T3, T4; Figure 5b).

CD55 is associated with src family protein tyrosine kinases p56lck and p59fyn.^23^ RAS-mediated Fyn upregulation results in decreased cell–cell adhesion and increased cell motility. In contrast, inhibition of Fyn activity results in increased cell–cell adhesion through the adherent junctions and blocked migratory capacity.^24^ We hypothesize that the absence of CD55 and consequently of the Fyn intracellular activated pathway implies an increased cell–cell adhesion of CD55^−^ cells. We analyzed the expression levels of Fyn protein (as determined by the integral optical densities, and normalized with respect to β-actin expression) in sorted (CD55^+^ and CD55^−^) and unsorted (CHP134 and IMR32) cell lines by western blotting (Figure 5c). The CD55^+^-sorted cells and the unsorted cells have major expression of Fyn protein compared with the CD55^−^-sorted cells. The similar Fyn activation in CD55+ cells and in unsorted cell lines might be explained by the paracrine effects mediated by secreted CD55. Our findings suggest that the Fyn downstream pathway determined by the presence of CD55 protein carries out anti-adhesive effects on NB cells.

### Correlation between *CD55* gene expression and prognosis in NB

**Figure 6:**
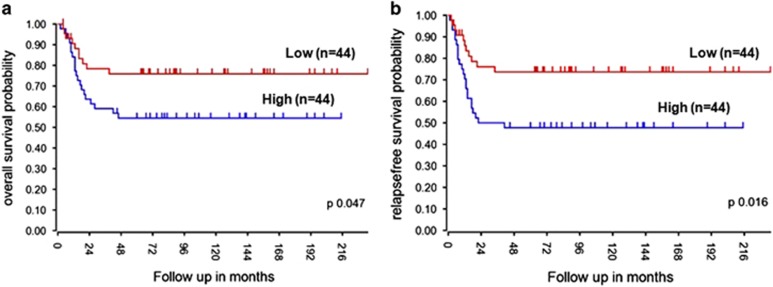
*CD55* gene expression is associated with poor survival in NB patients. Kaplan–Maier analysis is shown, with individuals grouped by the median of expression of *CD55* for overall survival (**a**) and relapse-free survival (**b**) rates in 88 NB patients (Versteeg data set).

The clinical significance of *CD55* gene expression in NB was investigated. We evaluated the association of *CD55* expression with clinical outcomes in NB patients using the Versteeg data set that is deposited in the R2 microarray web tool and includes 88 patients. As shown in Figure 6, high mRNA levels of *CD55* were significantly associated with lower overall survival and relapse-free survival (Figures 6a and b). When we restricted the analysis to patients with metastatic tumor (stage 4), high mRNA levels of *CD55* showed a trend for the association with overall and relapse-free survival but not in the subset of patients with localized tumors (stage 1–2–3; Supplementary Figure 6). These data show a significant deterioration in prognosis with increased *CD55* expression.

## Discussion

The upregulation of complement inhibitor factors (CD55, CD59 and CD46) is one of several strategies adopted by tumor cells to evade the immune system.^25, 26^ A number of secondary functions for complement regulatory proteins has been identified, including cell adhesion and being mediators of regulatory T cells.^27^ The mechanisms that result in their production and their role in the pathogenesis of tumors remain to be defined, although their overexpression potentially makes them a good therapeutic target.

A functional binding site for the transcriptional regulator hypoxia-inducible factors (HIFs) has been identified on the CD55 promoter.^20^ HIFs (HIF-1α, HIF-2α) have been shown to be regulators of the response to low oxygen concentration in various solid tumors and to be related to tumor malignancy.^28^ The well-known CD55 expression upon HIF stabilization in hypoxia and CD55 expression in solid tumors suggest CD55 as a novel HIF target that contributes to tumor defense mechanisms for survival in adverse physiological conditions.

CD55 and HIF-2α proteins have been shown to be independent indicators of poor prognosis in several tumors by immunohistological staining.^15, 17, 29, 30^ In this study we demonstrated that CD55 protein expression is limited to a small subset of tumor cells highly expressing HIF-2α in NB tumors. In the literature HIF-2α is correlated with aggressive and metastatic NB,^31^ and under non-hypoxic conditions creates a pseudo-hypoxic phenotype with clear influence on tumor behavior *in vivo*.^32^ Here we observed that high levels of CD55 are associated with bad survival in patients with stage 4 tumors but not in those with stage 1–2–3 tumors, suggesting that CD55 gene expression correlates mainly with tumor recurrence and metastasis.

HIF-2α has an important role in the regulation of stem cell maintenance within solid tumors ^7, 8^ but then HIF-2α targeting has limited application in clinics because of protein instability in oxygenated areas. The identification of CD55 as a HIF-2α marker might open up new therapeutic opportunities for the treatment of recurrence and metastasis. To dissect the role of CD55 as a HIF-2α marker, first we verified CD55 expression upon HIF-2α expression in NB cells *in vitro* and we found that CD55 is univocally regulated by HIF-2α. HIF-2α protein overexpression was evaluated in normoxic conditions as we have previously shown HIF-2α stability in normoxic NB cells.^22^ In NB cells HIF-2α expression is sufficient to enhance stem features, whereas CD55, although described as a putative marker of cancer stem cells,^19^ has not influenced the stem-like properties of NB cells so far *in vitro*. In contrast, CD55 promotes NB cell aggressiveness as cell growth and invasion upon HIF-2α expression.

To functionally assess the role of CD55 in NBs we verified the expression of CD55 in NB cell lines *in vitro*. CD55^high^ cell populations of breast cancer cells have been determined to correlate with increased ability to form colonies *in vitro* and with metastatic potential *in vivo*.^17^ For this reason we searched for CD55 antigen expression level in several NB cell lines, and, given the relatively low abundance of cells positive for CD55 (CD55^+^) as regards CD55-negative cells (CD55^−^), our aim has been to elucidate the role of this subpopulation in NB cell lines.

We verified that the CD55^+^ cell population displays the characteristics of an aggressive cancer, with decreased adhesion facilitating increased motility and invasion, coupled with the ability to survive and form colonies in anchorage-independent conditions. These features could be compared with the *in vivo* situation where the ability of tumor cells to detach from the primary tumor, invade through the ECM, survive in the blood stream, and form tumors at secondary sites leads to the formation of metastases. In contrast, the CD55^−^ cell population represents an *in vitro* model of tumor cells with decreased invasion, increased adhesion and reduced ability to grow and form colonies in anchorage-independent conditions. Moreover, CD55 protein is a soluble protein secreted by CD55^+^ cells, which might exercise a paracrine effect on CD55^−^ cells. Our hypothesis is that CD55^+^ cells in the heterogeneous cell population hold the invading ability of the entire heterogeneous cell line, thus activating the pathways for cell growth and invasion. Further investigation of this aggressive phenotype *in vivo* may help to define the CD55 target for invasion and metastasis in NB.

To date, despite recent improvements in survival in randomized trials, NB patient outcome remains poor.^33^ Recently we have shown that the combined treatment of HIF inhibition with a differentiating agent enhances NB differentiation in the more benign phenotype.^22^ This finding has underlined the impact of HIF signaling or HIF marker inhibition in NB therapy. In the present study we show that CD55 is a HIF-2α marker and has anti-adhesive and pro-invading functions that might provide the basis for NB solid tumors to survive as microscopic residual disease. Thanks to accessibility of the CD55 cell surface protein, this marker will provide a ready target for the monoclonal antibody in order to destroy a small cell population within tumors responsible for tumor recurrence. Antibodies against CD55 have been already used as additive therapeutic agents in the treatment of patients with breast and gastric cancer.^16, 34^

Furthermore, the use of CD55 antibody-based visualization as in PET (Positron Emission Tomography) imaging will have implications for the development of more accurate diagnosis and prognosis in challenging cases and for driving personalized treatment. The feasibility to test this new approach in carefully controlled NB clinical trials should finally result in the type of progress seen for breast cancer.^35^

## Material and methods

### Cell culture

The human SHSY5Y (American Type Culture Collection (ATCC) CRL-2266), SKNAS (ATCC CRL-2137), NMB (Deutsche Sammlung von Mikroorganismen und Zellkulturen (DSMZ) ACC 6579), NGP (DSMZ ACC 676) and SKNFI (ATCC CRL-2142) cell lines were grown in Dulbecco's modified Eagle's medium (DMEM); the human SKNBE2c (ATCC CRL-2268) cell line was grown in DMEM F-12; the human IMR32 (ATCC CCL-127) and SKNSH (ATCC HTB11) cell lines were grown in Minimum Essential Medium Eagle (MEM); the human CHP134 (Sigma, St Louis, MO, USA, 06122002) cell line was grown in RPMI-1640 Medium; the human NBLS (DSMZ ACC 656) cell line was grown in ISCOVE'S Medium. All media were supplemented with 10% heat-inactivated fetal bovine serum, 1 mM l-glutamine, penicillin (100 U/ml) and streptomycin (100 μg/ml), and the cells were grown at 37 °C, 5% CO_2_ in a humidified atmosphere. The cumulative culture length of the cells was fewer than 6 weeks after resuscitation. Early-passage cells were used for all experiments and they were not reauthenticated. All cell culture reagents were provided by Sigma-Aldrich (Milan, Italy).

### Flow cytometry

NB cell clones (HIF-2α, CD55 and pcDNA) and NB cell lines (3 × 10^5^ cells) were washed twice with phosphate-buffered saline (PBS) and incubated for 1 h at 4 °C with anti-human-CD55 phycoerythrin (PE)-coupled antibody (eBioscience 12-0559, San Diego, CA, USA). After incubation the cells were washed twice in PBS and resuspended in 500 μL of PBS. All tubes were acquired at Beckmann Coulter FC500 cytometer. For cell sorting, 2 × 10^6^ cells (CHP134, IMR32) were washed twice with PBS and incubated for 1 h at 4 °C with anti-human-CD55 PE-coupled antibody (eBioscience, San Diego, CA, USA). After incubation the cells were washed twice in PBS, resuspended in grown medium (4 ml) and sorted separately as CD55^+^ and CD55^−^ populations (BD FACSAria IIIu, San Jose, CA, USA). FACSDiva software (BD Bioscience, San Jose, CA, USA) was used for data analysis. Unstained cells were used as blank control to evaluate each antibody and cellular autofluorescence background (CTR).

### Colony formation assay in soft agar

NB cell clones (HIF-2α, CD55 and pcDNA) and CHP134, IMR32 (unsorted and CD55 sorted) cell lines were plated (2 × 10^5^ cells) in 0.35% agar on a bottom layer of 1% agar in 35-mm dishes of 6-well plates (Corning, New York, NY, USA). The plates were incubated at 37 °C for 4 weeks and stained with 0.01% Crystal violet. Colonies with 20 cells or more were counted. The means and standard deviations were calculated from three independent experiments.

### Invasion assay

To perform the invasion assay NB cell clones (HIF-2α, CD55 and pcDNA) and CHP134, IMR32 (unsorted and CD55 sorted) cell lines were trypsinized, counted (8 × 10^4^), suspended in 5% fetal bovine serum medium and seeded on the upper side of a transwell (8-μm pore size PET, Corning, NY, USA) that was previously coated with Collagene I (Sigma-Aldrich). The invasion toward the bottom of the insert driven by the presence of 15% fetal bovine serum was stopped after 12 h. The cells on the membrane were washed in PBS, fixed in acetic acid (5%) and ethanol (95%), and stained with Hematoxilin & Eosin (Sigma-Aldrich). The membrane images were acquired using a photocamera and the stained cells were counted using ImageJ software (Bethesda, MD, USA). The experiments were performed in triplicate and for each experiment three experimental points were analyzed.

### Adhesion assay

CHP134 and IMR32 (unsorted and CD55 sorted) cell lines were seeded (15 × 10^3^ cells) on a 96-multiwell previously coated with Collagen I or Fibronectin. At 0 (T0), 1 h (T1), 2 h (T2), 3 h (T3) and 4 h (T4) the cells were sucked from the wells and the remaining cells attached to the coated plates were visualized using the 3-(4,5-dimethylthiazol-2-yl),5-diphenyltetrazolium bromide assay, according to the manufacturer's protocol (Promega, Milan, Italy). The experiments were performed in triplicate, and for each experiment six experimental points were analyzed

### Immunohistochemistry

Clinical samples from the biobank of the Institute of Pathology of the University Hospital of Basel were used for the TMA construction as described in Supplementary Material and methods. Expression of HIF-2α and CD55 was analyzed by immunohistochemistry on 5-mm formalin-fixed paraffin-embedded sections of NB by using an antibody to HIF-2-α (Abcam ab8365, Cambridge, UK,) and to CD55 (HPA024386 Sigma-Aldrich). Antigen was retrieved by pretreating dewaxed sections in a microwave oven at 750 W for 5 min in citrate buffer (pH 6) and processing them with a Super Sensitive Link-Labeled Detection System (Biogenex, Florence, Italy). The enzymatic activity was developed using 3-amino-9-ethylcarbazole (AEC, Dako, Gostrup, Denmark) as achromogenic substrate. Following counterstaining with Mayer's haematoxylin, slides were mounted in aqueous mounting medium (glycergel, Dako, Gostrup, Denmark).

### Statistical analyses

All biochemical experiments were performed in triplicate unless otherwise stated. Two-tailed Student's *t*-test was used to test significance. Statistical significance was established at *P⩽0.05, **P⩽0.005 and ***P⩽0.0005. Survival curves were constructed by the Kaplan–Meier method, with differences between curves tested for statistical significance using the log-rank test.

## References

[bib1] Westermann F, Schwab M. Genetic parameters of neuroblastomas. Cancer Lett 2002; 184: 127–147. 12127685 10.1016/s0304-3835(02)00199-4

[bib2] Maris JM, Hogarty MD, Bagatell R, Cohn SL. Neuroblastoma. Lancet 2007; 369: 2106–2120. 17586306 10.1016/S0140-6736(07)60983-0

[bib3] Maris JM. Recent advances in neuroblastoma. N Engl J Med 2010; 362: 2202–2211. 20558371 10.1056/NEJMra0804577 PMC3306838

[bib4] Kamijo T, Nakagawara A. Molecular and genetic bases of neuroblastoma. Int J Clin Oncol 2012; 17: 190–195. 22588778 10.1007/s10147-012-0415-7

[bib5] Bhaskara VK, Mohanam I, Rao JS, Mohanam S. Intermittent hypoxia regulates stem-like characteristics and differentiation of neuroblastoma cells. PLoS ONE 2012; 7: e30905. 22363512 10.1371/journal.pone.0030905 PMC3281893

[bib6] De Miguel MP, Alcaina Y, de la Maza DS, Lopez-Iglesias P. Cell metabolism under microenvironmental low oxygen tension levels in stemness, proliferation and pluripotency. Curr Mol Med 2015; 15: 343–359. 25941818 10.2174/1566524015666150505160406

[bib7] Heddleston JM, Li Z, McLendon RE, Hjelmeland AB, Rich JN. The hypoxic microenvironment maintains glioblastoma stem cells and promotes reprogramming towards a cancer stem cell phenotype. Cell Cycle. 2009; 8: 3274–3284. 19770585 10.4161/cc.8.20.9701 PMC2825672

[bib8] Mohlin S, Hamidian A, Påhlman S. HIF2A and IGF2 expression correlates in human neuroblastoma cells and normal immature sympathetic neuroblasts. Neoplasia 2013; 15: 328–334. 23479510 10.1593/neo.121706 PMC3593155

[bib9] Macor P, Tedesco F. Complement as effector system in cancer immunotherapy. Immunol Lett 2007; 111: 6–13. 17572509 10.1016/j.imlet.2007.04.014

[bib10] Morgan J, Spendlove I, Durrant LG. The role of CD55 in protecting the tumour environment from complement attack. Tissue Antigens 2002; 60: 213–223. 12445304 10.1034/j.1399-0039.2002.600303.x

[bib11] Towner L, Donev R, Kolev M. Complement in cancer and cancer immunotherapy. Arch Immunol Ther Exp (Warsz) 2011; 59: 407–419. 21960413 10.1007/s00005-011-0146-x

[bib12] Loberg RD, Day LL, Dunn R, Kalikin LM, Pienta KJ. Inhibition of decay-accelerating factor (CD55) attenuates prostate cancer growth and survival *in vivo*. Neoplasia 2006; 8: 69–78. 16533428 10.1593/neo.05679 PMC1584292

[bib13] Hensel F, Timmermann W, von Rahden BH, Rosenwald A, Brändlein S, Illert B. Ten-year follow-up of a prospective trial for the targeted therapy of gastric cancer with the human monoclonal antibody PAT-SC1. Oncol Rep 2014; 31: 1059–1066. 24452482 10.3892/or.2014.2987 PMC3926647

[bib14] Mamidi S, Höne S, Teufel C, Sellner L, Zenz T, Kirschfink M. Neutralization of membrane complement regulators improves complement-dependent effector functions of therapeutic anticancer antibodies targeting leukemic cells. Oncoimmunology 2015; 4: e979688. 25949896 10.4161/2162402X.2014.979688 PMC4404820

[bib15] Shang Y, Chai N, Gu Y, Ding L, Yang Y, Zhou J et al. Systematic immunohistochemical analysis of the expression of CD46, CD55, and CD59 in colon cancer. Arch Pathol Lab Med 2014; 138: 910–919. 24978917 10.5858/arpa.2013-0064-OA

[bib16] Liu M, Yang YJ, Zheng H, Zhong XR, Wang Y, Wang Z et al. Membrane-bound complement regulatory proteins are prognostic factors of operable breast cancer treated with adjuvant trastuzumab: a retrospective study. Oncol Rep 2014; 32: 2619–2627. 25241923 10.3892/or.2014.3496

[bib17] Ikeda J, Morii E, Liu Y, Qiu Y, Nakamichi N, Jokoji R et al. Prognostic significance of CD55 expression in breast cancer. Clin Cancer Res 2008; 14: 4780–4786. 18676748 10.1158/1078-0432.CCR-07-1844

[bib18] Skelding KA, Barry RD, Shafren DR. Systemic targeting of metastatic human breast tumor xenografts by Coxsackievirus A21. Breast Cancer Res Treat 2009; 113: 21–30. 18256929 10.1007/s10549-008-9899-2

[bib19] Xu JX, Morii E, Liu Y, Nakamichi N, Ikeda J, Kimura H et al. High tolerance to apoptotic stimuli induced by serum depletion and ceramide in side-population cells: high expression of CD55 as a novel character for side-population. Exp Cell Res 2007; 313: 1877–1885. 17428472 10.1016/j.yexcr.2007.03.006

[bib20] Louis NA, Hamilton KE, Kong T, Colgan SP. HIF-dependent induction of apical CD55 coordinates epithelial clearance of neutrophils. FASEB J 2005; 19: 950–959. 15923405 10.1096/fj.04-3251com

[bib21] Holmquist-Mengelbier L, Fredlund E, Löfstedt T, Noguera R, Navarro S, Nilsson H et al. Recruitment of HIF-1alpha and HIF-2alpha to common target genes is differentially regulated in neuroblastoma: HIF-2alpha promotes an aggressive phenotype. Cancer Cell 2006; 10: 413–423. 17097563 10.1016/j.ccr.2006.08.026

[bib22] Cimmino F, Pezone L, Avitabile M, Acierno G, Andolfo I, Capasso M et al. Inhibition of hypoxia inducible factors combined with all-trans retinoic acid treatment enhances glial transdifferentiation of neuroblastoma cells. Sci Rep 2015; 5: 11158. 26057707 10.1038/srep11158 PMC4460899

[bib23] Shenoy-Scaria AM, Kwong J, Fujita T, Olszowy MW, Shaw AS, Lublin DM. Signal transduction through decay-accelerating factor. Interaction of glycosyl-phosphatidylinositol anchor and protein tyrosine kinases p56lck and p59fyn 1. J Immunol 1992; 149: 3535–3541. 1385527

[bib24] Fenton SE, Hutchens KA, Denning MF. Targeting Fyn in Ras-transformed cells induces F-actin to promote adherens junction-mediated cell-cell adhesion. Mol Carcinog 2014; 54: 1181–1193. 24976598 10.1002/mc.22190

[bib25] Pio R, Ajona D, Lambris JD. Complement inhibition in cancer therapy. Semin Immunol 2013; 25: 54–64. 23706991 10.1016/j.smim.2013.04.001 PMC3733085

[bib26] Kesselring R, Thiel A, Pries R, Fichtner-Feigl S, Brunner S, Seidel P et al. The complement receptors CD46, CD55 and CD59 are regulated by the tumour microenvironment of head and neck cancer to facilitate escape of complement attack. Eur J Cancer 2014; 50: 2152–2161. 24915776 10.1016/j.ejca.2014.05.005

[bib27] Liu J, Miwa T, Hilliard B, Chen Y, Lambris JD, Wells AD et al. The complement inhibitory protein DAF (CD55) suppresses T cell immunity *in vivo*. J Exp Med 2005; 201: 567–577. 15710649 10.1084/jem.20040863 PMC2213052

[bib28] Maynard MA, Ohh M. The role of hypoxia-inducible factors in cancer. Cell Mol Life Sci 2007; 64: 2170–2180. 17514355 10.1007/s00018-007-7082-2 PMC11138439

[bib29] Koukourakis MI, Giatromanolaki A, Sivridis E, Simopoulos C, Turley H, Talks K et al. Hypoxia-inducible factor (HIF1A and HIF2A), angiogenesis, and chemoradiotherapy outcome of squamous cell head-and-neck cancer. Oncol Lett 2015; 9: 793–797. 12128120 10.1016/s0360-3016(02)02848-1

[bib30] He Z, Wu H, Jiao Y, Zheng J. Expression and prognostic value of CD97 and its ligand CD55 in pancreatic cancer. Int J Radiat Oncol Biol Phys 2002; 53: 1192–1202. 25624904 10.3892/ol.2014.2751 PMC4301556

[bib31] Pietras A, Gisselsson D, Ora I, Noguera R, Beckman S, Navarro S et al. High levels of HIF-2alpha highlight an immature neural crest-like neuroblastoma cell cohort located in a perivascular niche. J Pathol 2008; 214: 482–488. 18189331 10.1002/path.2304

[bib32] Pietras A, Johnsson AS, Påhlman S. The HIF-2α-driven pseudo-hypoxic phenotype in tumor aggressiveness, differentiation, and vascularization. Curr Top Microbiol Immunol 2010; 345: 1–20. 20517717 10.1007/82_2010_72

[bib33] Øra I, Eggert A. Progress in treatment and risk stratification of neuroblastoma: impact on future clinical and basic research. Semin Cancer Biol 2011; 21: 217–228. 21798350 10.1016/j.semcancer.2011.07.002

[bib34] Macor P, Tripodo C, Zorzet S, Piovan E, Bossi F, Marzari R et al. *In vivo* targeting of human neutralizing antibodies against CD55 and CD59 to lymphoma cells increases the antitumor activity of rituximab. Cancer Res 2007; 67: 10556–10563. 17975000 10.1158/0008-5472.CAN-07-1811

[bib35] Tabouret-Viaud C, Botsikas D, Delattre BM, Mainta I, Amzalag G, Rager O et al. PET/MR in breast cancer. Semin Nucl Med 2015; 45: 304–321. 26050658 10.1053/j.semnuclmed.2015.03.003

