# Supplementary material for: CD55 is a HIF-2α marker with anti-adhesive and pro-invading properties in neuroblastoma
Source: Oncogenesis. 2016 Apr 4;5(4):e212–. doi: 10.1038/oncsis.2016.20 (PMC4848835; doi:10.1038/oncsis.2016.20)
Supplement: Supplementary Figures [file oncsis201620x1.doc]

**Supplementary Figure 1, Cimmino et al**

**
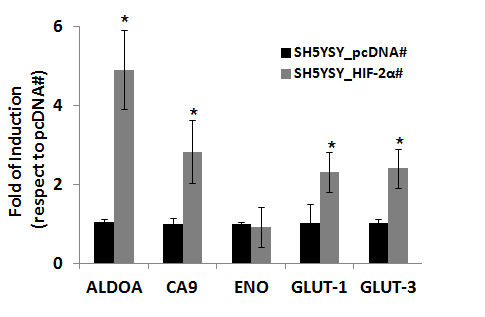
**

**Supplementary Figure 1. Hypoxia targets mRNA expression under HIF-2α induction.** The HIF-2α induction of the expression of hypoxia target genes as *ALDOA*, *CA9*, *ENO*, *GLUT-1*, *GLUT-3* was evaluated by RT-PCR in the SHSY5Y_HIF-2α# overexpressing clones and SHSY5Y_pcDNA# clones. The data are fold-changes of induction with respect to the SHSY5Y_pcDNA# cells. The mean fold change of 2^-(average ∆∆CT)^ was determined using the mean difference in the ∆C_T_ between the gene expression in the HIF-2α# cells and the ∆C_T_ for gene expression in the pcDNA# cells (as internal control). Data are means of three experiments (* P ≤0.05).

**Supplementary Figure 2, Cimmino et al**


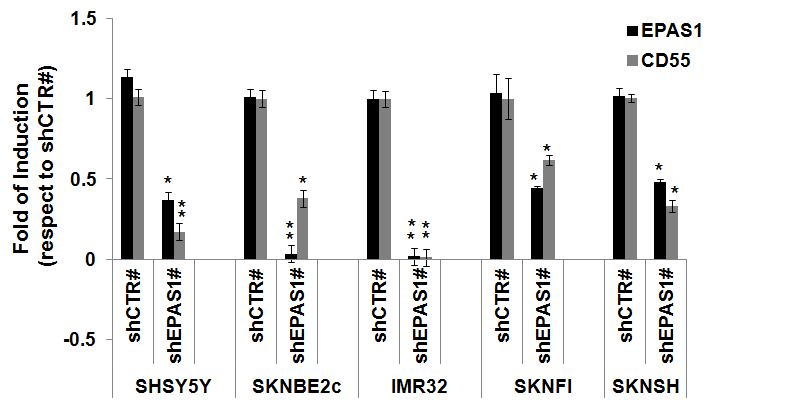


**Supplementary Figure 2. *CD55* mRNA expression under *EPAS1* (HIF-2α gene) silencing.** The *EPAS1* induction of the expression of *CD55* was evaluated by RT-PCR in the SHSY5Y, SKNBE2c, IMR32, SKNFI and SKNSH cell lines previously silenced for *EPAS1* expression (shEPAS1). The data are shown as fold-changes of induction with respect to the shCTR unsilenced cells. *EPAS1* mRNA silencing was also verified in the same cell lines. The mean fold change of 2^-(average ∆∆CT)^ was determined using the mean difference in the ∆C_T_ between the gene expression in the shEPAS1cells and the ∆C_T_ for gene expression in the shCTR cells (as internal control). Data are means of three experiments (* P ≤0.05; ** P ≤0.01).

**Supplementary Figure 3, Cimmino et al**


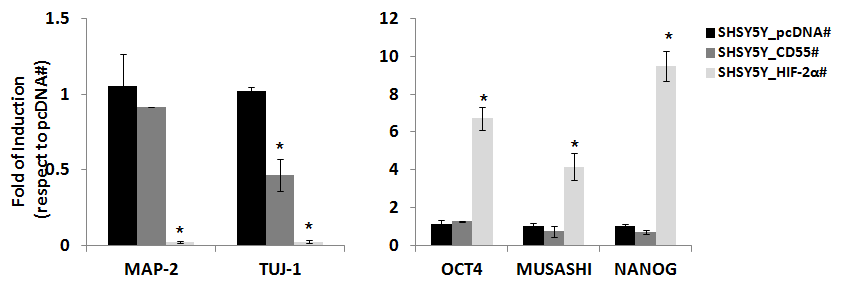


**Supplementary Figure 3. Neuronal and stem markers mRNA expression under HIF-2α and CD55 induction.** The HIF-2α and CD55 induction of the expression of neuronal markers as MAP-2 and TUJ-1, and of stem markers as OCT4, MUSASHI and NANOG was evaluated by RT-PCR in the SHSY5Y_pcDNA#, SHSY5Y_HIF-2α# and SHSY5Y_CD55# cell clones. The data are fold-changes of induction with respect to the pcDNA# cells. The mean fold change of 2^-(average ∆∆CT)^ was determined using the mean difference in the ∆C_T_ between the gene expression in HIF-2α# or CD55# cells and the ∆C_T_ for gene expression in the pcDNA# cells (as internal control). Data are means of three experiments (* P ≤0.05; ** P ≤0.01).

**Supplementary Figure 4, Cimmino et al**


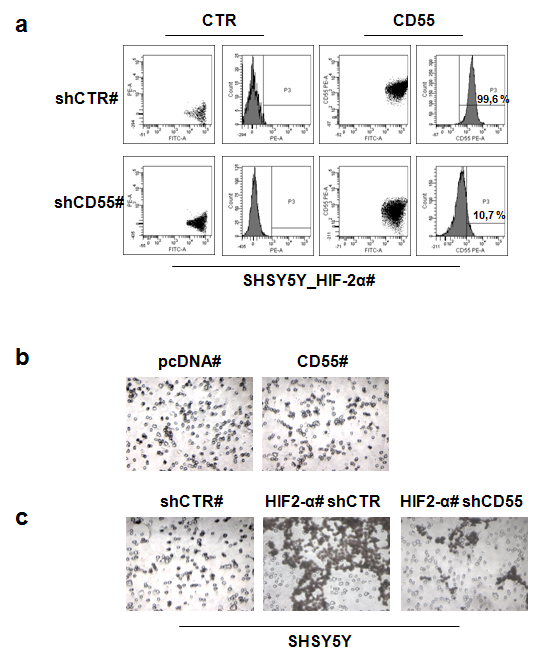


**Supplementary Figure 4. *CD55* silencing in SHSY5Y_HIF-2α# clones impairs cell invasion.** SHSY5Y_HIF-2α# cells were silenced for *CD55* expression (shCD55#) and SHSY5Y_HIF-2α# unsilenced were used as control (shCTR#). The decrement of CD55 protein on the surface membrane of silenced SHSY5Y_HIF-2α# cells (shCD55#) is shown by FACS staining. Contrary CD55 is positive in SHSY5Y_HIF-2α# unsilenced cells (shCTR#) (a). Invasive ability of SHSY5Y_CD55# cells and SHSY5Y_pcDNA# cells was evaluated as shown in photos (b). Invasive ability of SHSY5Y_HIF-2α# cells CD55 silenced (shCD55#) and unsilenced (shCTR) was evaluated as shown in photos (c). The data shown are representative of three independent experiments.

**Supplementary Figure 5, Cimmino et al**

**
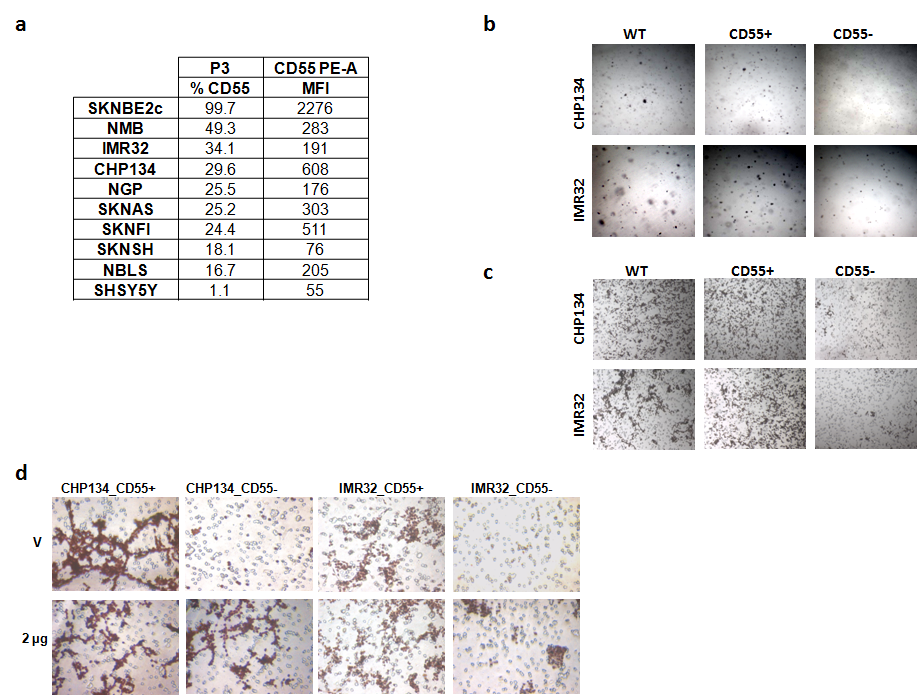
**

**Supplementary Figure 5. CD55 positive cells (CD55^+^) show enhanced soft agar growth and invasive ability compared to CD55 negative cells (CD55^-^) in NB cell lines.** CD55 positive cells subpopulation was identified in several NB cell lines by FACS cell surface staining. The % CD55 positivity and the CD55 mean fluorescence intensity (MFI) values are reported for all the analyzed NB cell lines (a). The soft agar growth (b) and the invasion ability (c) of CD55 positive cells (CD55^+^) and CD55 negative cells (CD55^-^) previously sorted by FACS were evaluated compared to unsorted cell lines (CHP134, IMR32). Invasion assay was performed on CD55^+^ and CD55^-^ sorted cells (CHP134 or IMR32) in presence of 2µg human recombinant protein CD55 or vehicle (d). The data shown are representative of three independent experiments.

**Supplementary Figure 6, Cimmino et al**


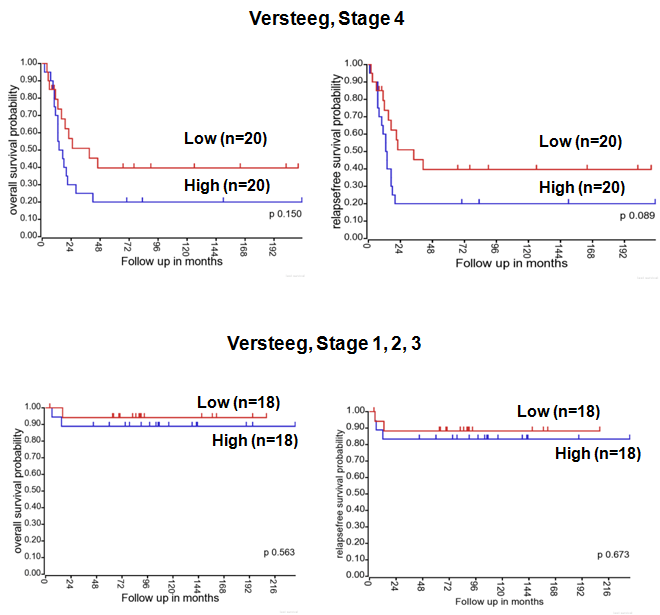


**Supplementary Figure 6. *CD55* expression is associated with poor survival for patients with stage 4 NB.** Kaplan-Maier analysis with patients grouped according to the median (calculated using the R2 web tool) in the expression of *CD55* for overall survival and relapse-free survival for 40 patients with stage 4 NB and for 36 patients with stage 1, 2, 3 NB (Versteeg dataset).
