# Supplementary material for: CD55 is a HIF-2α marker with anti-adhesive and pro-invading properties in neuroblastoma
Source: Oncogenesis. 2016 Apr 4;5(4):e212–. doi: 10.1038/oncsis.2016.20 (PMC4848835; doi:10.1038/oncsis.2016.20)
Supplement: Supplementary Information [file oncsis201620x2.doc]

**Supplementary Material and Methods**

**Cloning and stable clones generation**

A 2.7 kb human HIF-2α cDNA was cloned into the *NOT1-XbaI*site of pcDNA B vector ( Invitrogen™ life technologies) to obtain the in-frame coding sequence with Flag epitote. A 1.1 kb human CD55 cDNA was cloned into the *NOT1-XbaI*site of pcDNA3.1 vector ( Invitrogen™ life technologies). The cloning sequences were confirmed by DNA sequencing and the protein expression were validated by western blotting.

SHSY5Y cell lines were transfected with 12μg of pcDNA-B (used as control) or pcDNA-HIF-2α or pcDNA-CD55 using Transfectin™ Lipid Reagent (BIO-RAD). Forty-eight hours after transfection, G418 (0,5 μg/ml, Euroclone) was added to the medium for the selection of transfected cells. After 40-day selection with G418, surviving SHSY5Y cell colonies were isolated and repeatedly propagated in a large scale in the same medium with 0,5 µg/ml of G-418 per for further analyses. Several clones were verified for protein and gene expressions. The experiments have been repeated for three different clones in triplicate.

**Western Blotting**

For supernatant protein extraction the cells were grown in medium without serum. After 24hours the medium were collected in Amicon Ultra-15 (Millpore) and centrifuge at 10000 rpm for 30 minutes. For total protein extract the cells were lysed in protein lysis buffer (20 mMsodium phosphate, pH 7.4, 150 mMNaCl, 10% glycerol, 1% Na-deoxycholate and 1% Triton X-100) supplemented with protease inhibitors (Roche).Protein concentrations were determined by Bradford Assay (Bio-Rad). Thirty micrograms of protein were electrophoresed on 8% SDS-PAGE gels and

transferred onto PVDF membranes (Bio-Rad). After 1 h blocking with 5% dry milk fat in phosphate-buffered saline (PBS) containing 0.02% Tween-20, the membranes were incubated with the primary antibody overnight at 4°C, and with the secondary antibody for 1 h at room temperature. The bands were visualized with a chemiluminescence detection system (Pierce), according to the manufacturer instructions. The antibodies used were as follows: anti-Flag (Sigma), anti-HIF-2α (Abcam ab8365), anti-CD55 (**HPA024386** Sigma), anti-Fyn59 (GTX109428 GeneTex), Anti-β-actin (**A3854** Sigma). The protein bands image were acquired with GelDoc 2000 system (Bio-Rad).

**Production of Lentiviral particles and Infection of cell lines**

To knockdown CD55 or EPAS1 gene expression, pGIPZ Lentiviral shRNAmir targeting human CD55 or EPAS1 were purchased from Open Biosystems (Thermo Fisher Scientific, Inc.). We used three different shRNA for *CD55*: V2LH228269 (RHS4430-200187910), V2LHS-111497 (RHS4430-200198427) and V2LHS-111499 (RHS4430-200209398). We used two different shRNA for *EPAS1*: V2LHS113750 (RHS4430-98894439) and V2LHS-113750 (RHS4430-98851126). A non-silencing pGIPZ Lentiviral shRNAmir was used as control (RHS4346). The lentivirus production and lentiviral production was performed as described (REF). The reported data are representative of the experiments performed and confirmed by using all three lentiviral vectors for CD55.

**Tissue microarrays**

A Tissue microarray containing 92 neuroblastoma tumor samples was used in this study. Tissue samples were retrieved from the archives of the Institute of Pathology (University of Basel, Switzerland) and were reviewed by experienced pathologists (L.T. and L.M.T.). Briefly, to construct the TMAs, tissue samples were fixed in buffered 4 % formalin and embedded in paraffin. H&E-stained sections were made from each selected primary block (named donor blocks) to define representative tissue regions. Tissue cylinders (0.6 mm in diameter) were then punched from the region of the donor block with the use of a custom-made precision instrument (Beecher Instruments, Silver Spring, USA). Afterwards, tissue cylinders were transferred to a 25 x 35 mm paraffin block to produce the TMA. The resulting TMA block was cut into 3-lm sections that were transferred to glass slides by use of the paraffin sectioning aid system (Instrumedics, Hackensack, USA). Sections from the TMA blocks were used for immunoistochemistry analysis. The use of the clinical samples from the biobank of the Institute of Pathology for the TMA construction was approved by the Ethics Committee of the University Hospital of Basel (EKBB).

### Real-time RT-PCR

The expression levels of several genes were analyzed using real-time, quantitative PCR. Total RNA extraction using TRIzol LS Reagent (Invitrogen) and cDNA retrotranscription using the High Capacity cDNA Reverse Transcription Script (Applied Biosistem) was performed according to the manufacturer protocol. The cDNA samples were diluted to 20 ng/μl. Gene-specific primers were designed by using PRIMEREXPRESS software (Applied Biosystems), as: ALDOA (F) GACAGCTGACGACCGCGT, (R) CCATCATCCGCCTTCTGGTA, CAXI (F) TGCTAAGCAGCTCCACACCC, (R) TGCGTCGCTCGGAAGTTC; ENO (F) CCCGTGGTGAACGAGTGACT, (R) TGAAACCCAGTGTTCCATTCTG; GLUT1 (F) CCTGCTCATCAACCGCAAC, (R) TCATGGGTCACGTCAGCTGT; GLUT3 (F) CCAGCTGGGCATCGTTG (R) CGGCCATAGCTCTTCAGACC; EPAS1 (F) GACCCAAGATGGCGACATG, (R) TGTCCTGTTAGCTCCACCTGTG; CD55 (F) CCCGTTGCCAGAGTGCAG (R) TGGTCACGTTCCCCTTGAAT; TUJ-1 (F) TATGAGGGAGATCGTGCACATC, (R) TGACTTCCCAGAACTTGGCC; MAP-2 (F) TCTCTTCTTCAGCGCACCGGCG, (R); OCT4 (F) ACTGCAGCAGATCAGCCACA ® TGGCGCCGGTTACAGAAC, Musashi (F) CCCCTGACCAAGAGATCCAG ® CGATTGCGCCAGCACTTT; NANOG (F) GAACCTCAGCTACAAACAGGTGAA ® GGCCAGTTGTTTTTCTGCCA ® β-Actin (F) CGTGCTGCTGACCGAGG, (R) GAAGGTCTCAAACATGATCTGGGT. Real-time PCR was performed using SYBR Green PCR Master Mix (AppliedBiosystems). All real-time PCR reactions were performed using the 7900HT Fast Real-Time PCR System (Applied Biosystems). The experiments were carried out in triplicate for each data point. The housekeeping gene β-actin was used as the internal control. Relative gene expression was calculated using the 2^-ΔCT^ method, where the ∆C_T_ was calculated using the differences in the mean C_T_ between the selected genes and the internal control (β-actin). The mean fold change of 2^-(average ∆∆CT)^ was determined using the mean difference in the ∆C_T_ between the gene of interest and the internal control.

**Cloning and stable clones generation**

A 2.7 kb human HIF-2α cDNA was cloned into the *NOT1-XbaI*site of pcDNA B vector ( Invitrogen, Milan, Italy) to obtain the in-frame coding sequence with Flag epitote. A 1.1 kb human CD55 cDNA was cloned into the *NOT1-XbaI*site of pcDNA3.1 vector ( Invitrogen, Milan, Italy). The cloning sequences were confirmed by DNA sequencing and the protein expression were validated by western blotting.

SHSY5Y cell lines were transfected with 12μg of pcDNA-B (used as control) or pcDNA-HIF-2α or pcDNA-CD55 using Transfectin™ Lipid Reagent (Bio-Rad, Milan, Italy). Forty-eight hours after transfection, G418 (0,5 μg/ml, Euroclone, Milan, Italy) was added to the medium for the selection of transfected cells. After 40-day selection with G418, surviving SHSY5Y cell colonies were isolated and repeatedly propagated in a large scale in the same medium with 0,5 µg/ml of G-418 per for further analyses. Several clones were verified for protein and gene expressions. The experiments have been repeated for three different clones in triplicate.

**Western Blotting**

For supernatant protein extraction the cells were grown in medium without serum. After 24hours the medium were collected in Amicon Ultra-15 (Millpore, Cork, Ireland) and centrifuge at 10000 rpm for 30 minutes. For total protein extract the cells were lysed in protein lysis buffer (20 mMsodium phosphate, pH 7.4, 150 mMNaCl, 10% glycerol, 1% Na-deoxycholate and 1% Triton X-100) supplemented with protease inhibitors (Roche, Milan, Italy).Protein concentrations were determined by Bradford Assay (Bio-Rad, Milan, Italy). Thirty micrograms of protein were electrophoresed on 8% SDS-PAGE gels and

transferred onto PVDF membranes (Bio-Rad, Milan, Italy). After 1 h blocking with 5% dry milk fat in phosphate-buffered saline (PBS) containing 0.02% Tween-20, the membranes were incubated with the primary antibody overnight at 4°C, and with the secondary antibody for 1 h at room temperature. The bands were visualized with a chemiluminescence detection system (Pierce), according to the manufacturer instructions. The antibodies used were as follows: anti-Flag (Sigma), anti-HIF-2α (Abcam, [Cambridge](https://en.wikipedia.org/wiki/Cambridge), UK,), anti-CD55 (Sigma-Aldrich, Milan, Italy), anti-Fyn59 (GeneTex, CA, USA), Anti-β-actin (Sigma-Aldrich Milan, Italy). The protein bands image were acquired with GelDoc 2000 system (Bio-Rad, Milan, Italy).

**Production of Lentiviral particles and Infection of cell lines**

To knockdown CD55 gene expression, pGIPZ Lentiviral shRNAmir targeting human CD55 were purchased from Open Biosystems (Thermo Fisher Scientific, MA, USA). We used three different shRNA for CD55: V2LH228269 (RHS4430-200187910), V2LHS-111497 (RHS4430-200198427) and V2LHS-111499 (RHS4430-200209398). A non-silencing pGIPZ Lentiviral shRNAmir was used as control (RHS4346). The lentivirus production and lentiviral production was performed as described (REF). The reported data are representative of the experiments performed and confirmed by using all three lentiviral vectors for CD55.

**Tissue microarrays**

A Tissue microarray containing 92 neuroblastoma tumor samples was used in this study. Tissue samples were retrieved from the archives of the Institute of Pathology (University of Basel, Switzerland) and were reviewed by experienced pathologists (L.T. and L.M.T.). Briefly, to construct the TMAs, tissue samples were fixed in buffered 4 % formalin and embedded in paraffin. H&E-stained sections were made from each selected primary block (named donor blocks) to define representative tissue regions. Tissue cylinders (0.6 mm in diameter) were then punched from the region of the donor block with the use of a custom-made precision instrument (Beecher Instruments, Silver Spring, MD, USA). Afterwards, tissue cylinders were transferred to a 25 x 35 mm paraffin block to produce the TMA. The resulting TMA block was cut into 3-lm sections that were transferred to glass slides by use of the paraffin sectioning aid system (Instrumedics, Hackensack, NJ, USA). Sections from the TMA blocks were used for immunoistochemistry analysis. The use of the clinical samples from the biobank of the Institute of Pathology for the TMA construction was approved by the Ethics Committee of the University Hospital of Basel (EKBB).

### Real-time RT-PCR

The expression levels of several genes were analyzed using real-time, quantitative PCR. Total RNA extraction using TRIzol LS Reagent (Invitrogen, MA, USA) and cDNA retrotranscription using the High Capacity cDNA Reverse Transcription Script (Applied Biosistem, MA, USA) was performed according to the manufacturer protocol. The cDNA samples were diluted to 20 ng/μl. Gene-specific primers were designed by using PRIMEREXPRESS software (Applied Biosystems, MA, USA), as: ALDOA (F) GACAGCTGACGACCGCGT, (R) CCATCATCCGCCTTCTGGTA, CAXI (F) TGCTAAGCAGCTCCACACCC, (R) TGCGTCGCTCGGAAGTTC; ENO (F) CCCGTGGTGAACGAGTGACT, (R) TGAAACCCAGTGTTCCATTCTG; GLUT1 (F) CCTGCTCATCAACCGCAAC, (R) TCATGGGTCACGTCAGCTGT; GLUT3 (F) CCAGCTGGGCATCGTTG (R) CGGCCATAGCTCTTCAGACC; EPAS1 (F) GACCCAAGATGGCGACATG, (R) TGTCCTGTTAGCTCCACCTGTG; CD55 (F) CCCGTTGCCAGAGTGCAG (R) TGGTCACGTTCCCCTTGAAT; TUJ-1 (F) TATGAGGGAGATCGTGCACATC, (R) TGACTTCCCAGAACTTGGCC; MAP-2 (F) TCTCTTCTTCAGCGCACCGGCG, (R); OCT4 (F) ACTGCAGCAGATCAGCCACA ® TGGCGCCGGTTACAGAAC, Musashi (F) CCCCTGACCAAGAGATCCAG ® CGATTGCGCCAGCACTTT; NANOG (F) GAACCTCAGCTACAAACAGGTGAA ® GGCCAGTTGTTTTTCTGCCA ® β-Actin (F) CGTGCTGCTGACCGAGG, (R) GAAGGTCTCAAACATGATCTGGGT. Real-time PCR was performed using SYBR Green PCR Master Mix (Applied Biosystems, MA, USA). All real-time PCR reactions were performed using the 7900HT Fast Real-Time PCR System (Applied Biosystems, MA, USA). The experiments were carried out in triplicate for each data point. The housekeeping gene β-actin was used as the internal control. Relative gene expression was calculated using the 2^-ΔCT^ method, where the ∆C_T_ was calculated using the differences in the mean C_T_ between the selected genes and the internal control (β-actin). The mean fold change of 2^-(average ∆∆CT)^ was determined using the mean difference in the ∆C_T_ between the gene of interest and the internal control.
